# Supplementary figures and images for: Longitudinal changes in glycemic control and associated factors in patients with type 2 diabetes mellitus in a public referral hospital in Peru
Source: PLoS One. 2026 Apr 6;21(4):e0346081. doi: 10.1371/journal.pone.0346081 (PMC13052837; doi:10.1371/journal.pone.0346081)

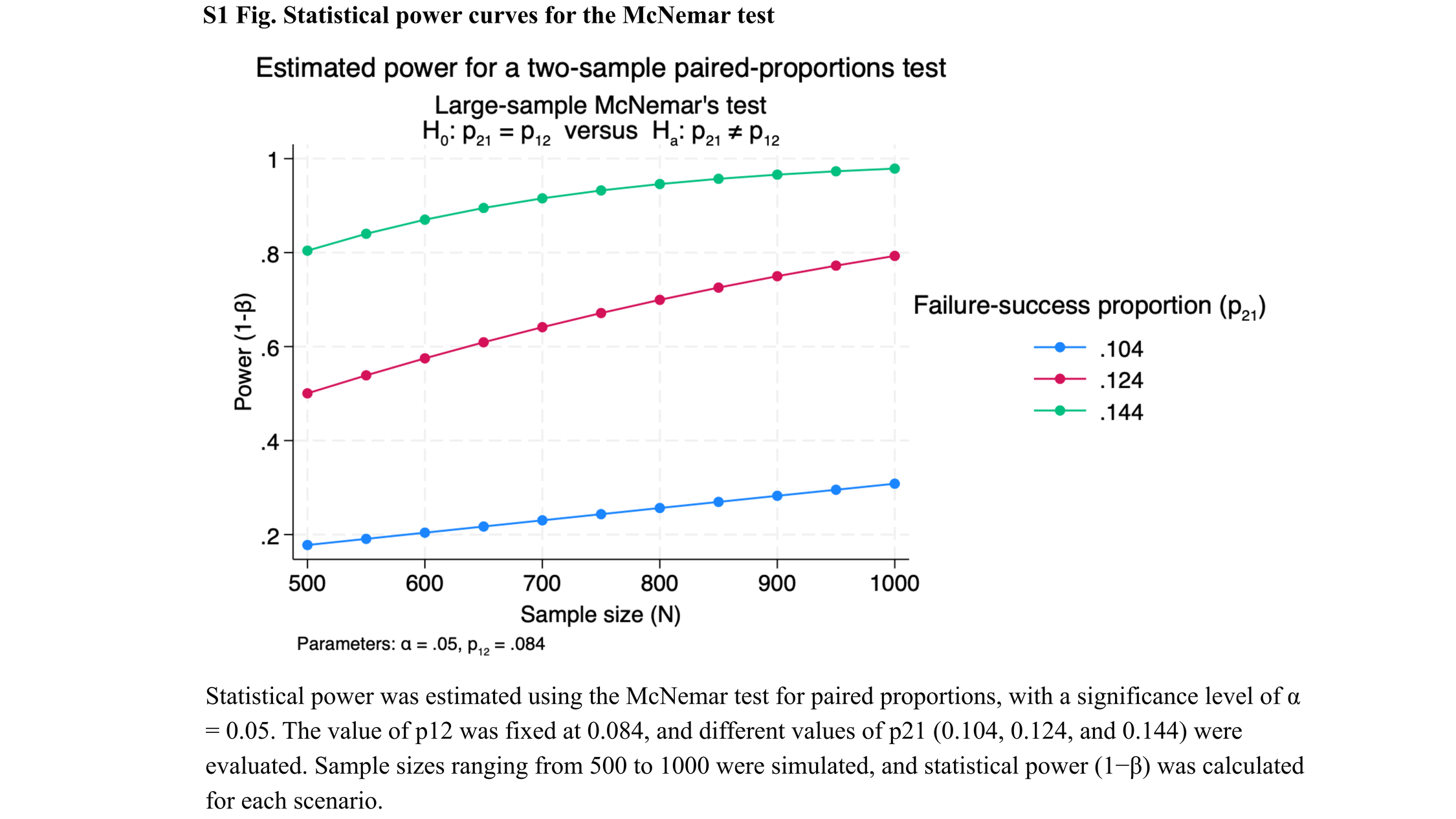

Supplement: S1 Fig — (TIF) [file pone.0346081.s001.tif]

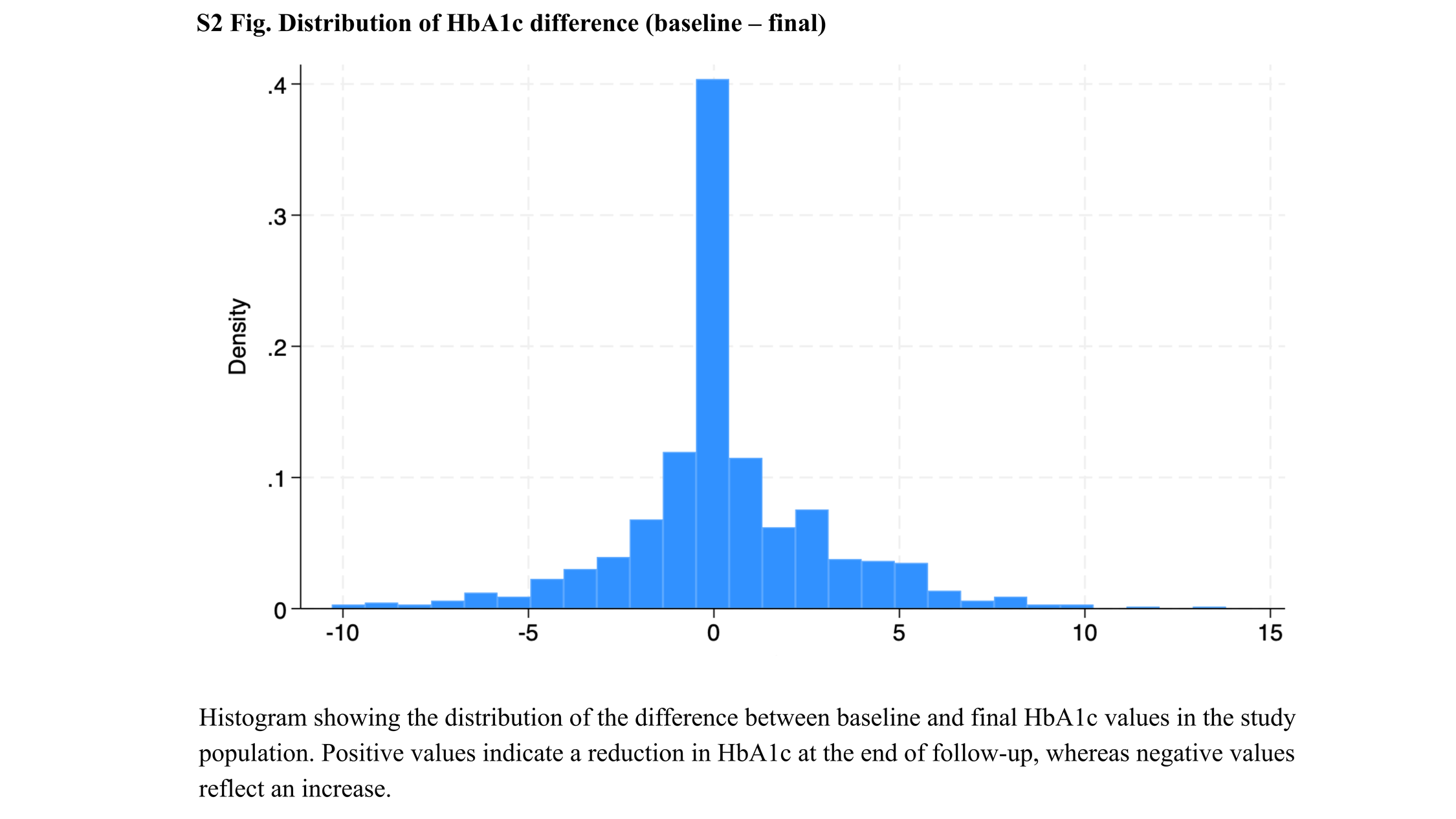

Supplement: S2 Fig — (TIF) [file pone.0346081.s002.tif]
