# Supplementary material for: Longitudinal changes in glycemic control and associated factors in patients with type 2 diabetes mellitus in a public referral hospital in Peru
Source: PLoS One. 2026 Apr 6;21(4):e0346081. doi: 10.1371/journal.pone.0346081 (PMC13052837; doi:10.1371/journal.pone.0346081)
Supplement: S3 Fig — (DOCX) [file pone.0346081.s003.docx]

**S3 Fig. Transition of HbA1c categories between baseline and final evaluation**


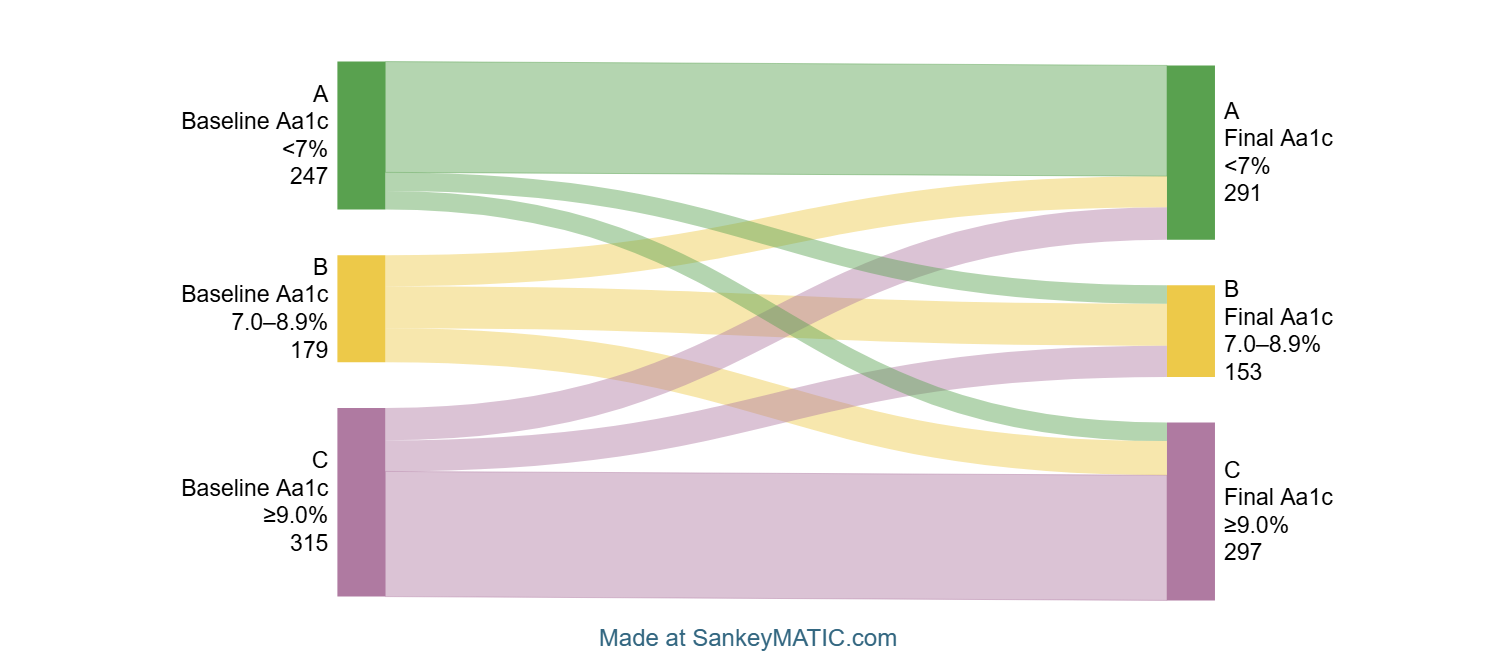


Sankey diagram illustrating the transition of patients between HbA1c categories (<7%, 7.0–8.9%, ≥9.0%) from baseline to final evaluation. Numbers indicate the frequency of patients in each category.
