# Supplementary material for: Longitudinal changes in glycemic control and associated factors in patients with type 2 diabetes mellitus in a public referral hospital in Peru
Source: PLoS One. 2026 Apr 6;21(4):e0346081. doi: 10.1371/journal.pone.0346081 (PMC13052837; doi:10.1371/journal.pone.0346081)
Supplement: S4 Fig — (DOCX) [file pone.0346081.s004.docx]

**S4 Fig. Baseline HbA1c and absolute change at one year**


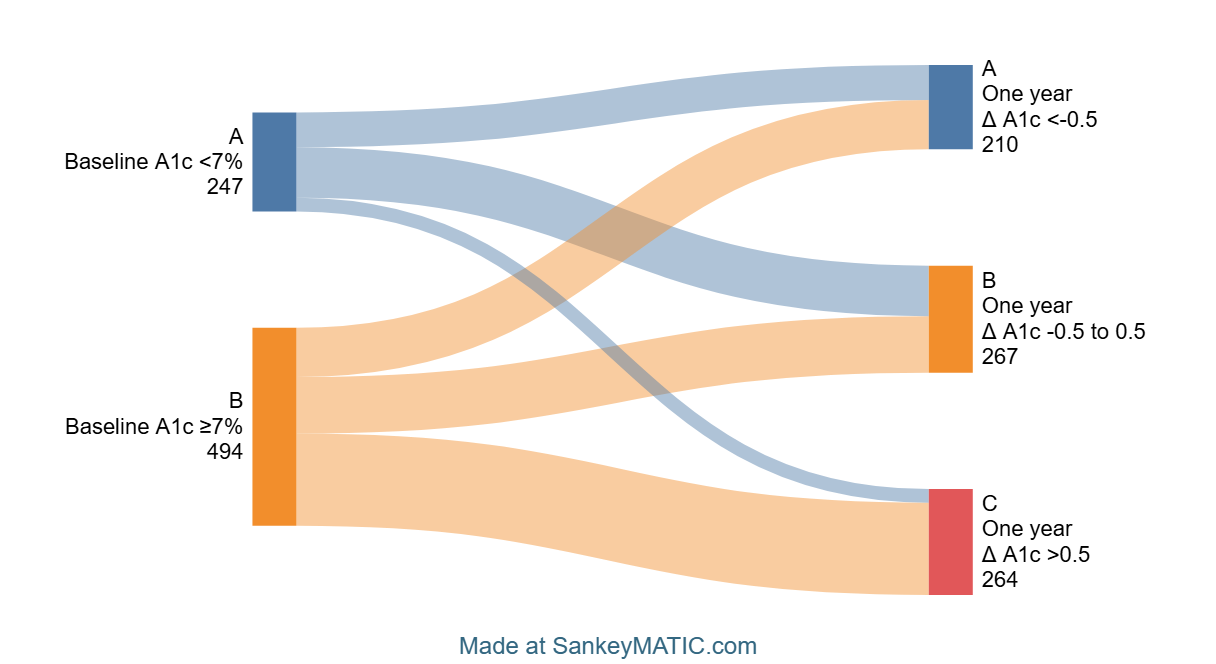


Sankey diagram showing patient transitions according to baseline HbA1c (<7% vs ≥7%) and absolute change in HbA1c (< −0.5%, −0.5% to 0.5%, > 0.5%) at one-year follow up.
