# Supplementary material for: Longitudinal changes in glycemic control and associated factors in patients with type 2 diabetes mellitus in a public referral hospital in Peru
Source: PLoS One. 2026 Apr 6;21(4):e0346081. doi: 10.1371/journal.pone.0346081 (PMC13052837; doi:10.1371/journal.pone.0346081)
Supplement: S1 Table — (DOCX) [file pone.0346081.s005.docx]

**S1 Table. Categories of HbA1c difference (ΔHbA1c) between baseline and final evaluation**

| Δ HbA1c (baseline – final) | n(%) |
| --- | --- |
| ≥ 4.00 | 44 (5.9) |
| 3.00 to 3.99 | 20 (2.7) |
| 2.00 to 2.99 | 29 (3.9) |
| 1.00 to 1.99 | 65 (8.8) |
| 0.50 to 0.99 | 52 (7.0) |
| -0.50 to 0.49 | 267 (36.0) |
| -1.00 to -0.51 | 53 (7.1) |
| -2.00 to -1.01 | 58 (7.8) |
| -3.00 to -2.01 | 52 (7.0) |
| -4.00 to -3.01 | 29 (3.9) |
| ≤-4.00 | 72 (9.2) |

HbA1c difference was calculated as baseline value minus final value. Positive values indicate a reduction in HbA1c (improvement in glycemic control), whereas negative values represent an increase in HbA1c (worsening of glycemic control).
