# Supplementary material for: Longitudinal changes in glycemic control and associated factors in patients with type 2 diabetes mellitus in a public referral hospital in Peru
Source: PLoS One. 2026 Apr 6;21(4):e0346081. doi: 10.1371/journal.pone.0346081 (PMC13052837; doi:10.1371/journal.pone.0346081)
Supplement: S2 Table — (DOCX) [file pone.0346081.s006.docx]

**S2 Table. Transition matrix of HbA1c categories (baseline vs final)**

| Baseline A1c | Final A1c < 7% | Final A1c 7 a 8.9% | Final A1c ≥ 9.0% |
| --- | --- | --- | --- |
| < 7% | 185 (24.9) | 31(4.2) | 31(4.2) |
| 7 a 8.9% | 52 (7.0) | 70 (9.5) | 57 (7.7) |
| ≥ 9.0% | 54 (7.3) | 52 (7.0) | 209 (28.2) |

Absolute frequencies and percentages of patients are shown according to baseline (rows) and final (columns) HbA1c categories. Categories were defined as <7%, 7.0–8.9% y ≥9.0%.
