# Supplementary material for: Longitudinal changes in glycemic control and associated factors in patients with type 2 diabetes mellitus in a public referral hospital in Peru
Source: PLoS One. 2026 Apr 6;21(4):e0346081. doi: 10.1371/journal.pone.0346081 (PMC13052837; doi:10.1371/journal.pone.0346081)
Supplement: S3 Table — (DOCX) [file pone.0346081.s007.docx]

**S3 Table. Factors associated with poor glycemic control (HbA1c ≥7%) in patients with type 2 diabetes according to different GEE model specifications**

| Variable / Category | Model 1 OR (95% CI) | p | Model 2 OR (95% CI) | p | Model 3 OR (95% CI) | p |
| --- | --- | --- | --- | --- | --- | --- |
| **Time (final vs baseline)** | **0.74 (0.62–0.88)** | **0.001** | **0.74 (0.63–0.88)** | **0.001** | **0.73 (0.61 -0.88)** | **0.001** |
| **Sex** |  |  |  |  |  |  |
| Female (ref.) | 1.00 |  | 1.00 | — | 1.00 |  |
| Male | 1.03 (0.76–1.39) | 0.834 | 1.03 (0.75–1.41) | 0.839 | 0.77 (0.57 – 1.05) | 0.104 |
| **Age** |  |  |  |  |  |  |
| <60 years (ref.) | 1.00 |  | 1.00 | — | 1.00 |  |
| ≥60 years | **0.73 (0.55–0.98)** | **0.042** | 0.74 (0.54–1.00) | 0.052 | 1.01 (0.73 – 1.39) | 0.944 |
| Educational level |  |  |  |  |  |  |
| Primary or less (ref.) | — | — | 1.00 | — | 1.00 |  |
| Secondary or higher | — | — | 1.07 (0.79–1.44) | 0.641 | 1.12 (0.31 – 1.53) | 0.439 |
| Missing data | — | — | 1.34 (0.83–2.15) | 0.225 | 1.41 (0.88 – 2.29) | 0.153 |
| Duration of diabetes |  |  |  |  |  |  |
| <10 years (ref.) | 1.00 |  | 1.00 | — | 1.00 |  |
| ≥10 years | **2.53 (1.80 – 3.56)** | **<0.001** | **2.54 (1.80-3.58)** | **<0.001** | 2.58 (1.82 – 3.64) | **<0.001** |
| Hypertension |  |  |  |  |  |  |
| No (ref.) | 1.00 | — | 1.00 | — | 1.00 |  |
| Yes | 0.98 (0.71–1.435) | 0.920 | 0.99 (0.72 – 1.36) | 0.960 | 1.07 (0.77 – 1.48) | 0.689 |
| BMI |  |  |  |  |  |  |
| <30 kg/m^2^ (ref.) | — | — | 1.00 | — | 1.00 |  |
| ≥30 kg/m^2^ | — | — | 0.95 (0.70–1.29) | 0.493 | 0.91 (0.67 – 1.23) | 0.531 |
| Missing data | — | — | 1.25 (0.49 -3.17) | 0.861 | 1.09 (0.38 – 3.08) | 0.689 |
| Diabetes treatment regimen |  |  |  |  |  |  |
| None | 0.96 (0.64-1.46) | 0.874 | 0.95 (0.62 – 1.45) | 0.834 | 0.94 (0.61 – 1.45) | 0.794 |
| Only OADs (ref.) | 1.00 |  | 1.00 |  | 1.00 |  |
| Insulin only | **5.51 (3.36 – 9.702)** | **<0.001** | 5.48 (3.34 – 9.01) | **<0.001** | **5.99 (3.65 – 9.82)** | **<0.001** |
| Insulin plus OADs | **4.91 (2.86 – 8.43)** | **<0.001** | **4.94 (2.87 – 8.51)** | **<0.001** | **5.19 (3.08 – 8.77)** | **<0.001** |
| Triglycerides |  |  |  |  |  |  |
| Normal (ref.) | — | — | — | — | 1.00 |  |
| Elevated | — | — | — | — | **2.06 (1.46 – 2.89)** | **<0.001** |
| Missing data | — | — | — | — | **1.58 (1.11 – 2.23)** | **0.010** |
| **eGFR** |  |  |  |  |  |  |
| ≥60 mL/min/1.73 m^2^ (ref.) | — | — | — | — | 1.00 |  |
| <60 mL/min/1.73 m^2^ | — | — | — | — | 0.54 (0.30 – 0.96) | 0.038 |
| Missing data | — | — | — | — | 0.87 (0.64 – 1.18) | 0.364 |

Model 1 includes only variables with complete data. Model 2 additionally incorporates educational level and BMI (approximately 10% missing data). Model 3 further includes variables with up to 35% missing data (triglycerides and eGFR). In Models 2 and 3, missing values were included as an explicit category (“Missing data”) to preserve sample size. This approach represents a pragmatic but methodologically imperfect solution that may introduce bias into the estimates.

BMI: body mass index; OAD: Oral antidiabetic drugs only ; eGFR:Estimated Glomerular filtration rate
