# Supplementary material for: Longitudinal changes in glycemic control and associated factors in patients with type 2 diabetes mellitus in a public referral hospital in Peru
Source: PLoS One. 2026 Apr 6;21(4):e0346081. doi: 10.1371/journal.pone.0346081 (PMC13052837; doi:10.1371/journal.pone.0346081)
Supplement: S4 Table — (DOCX) [file pone.0346081.s008.docx]

**S4 Table. Sensitivity analysis using GEE models restricted to complete cases for the assessment of factors associated with poor glycemic control (HbA1c ≥7%) between baseline and final evaluation**

| Variable / Category | Model 1 OR (95% CI) | p | Model 2 OR (95% CI) | p | Model 3 OR (95% CI) | p |
| --- | --- | --- | --- | --- | --- | --- |
| Sample size | 741 |  | 665 |  | 312 |  |
| Time (final vs baseline) | **0.74 (0.62–0.88)** | **0.001** | **0.76 (0.63–0.90)** | **0.002** | 0.77 (0.58 **–**1.01) | 0.055 |
| **Sex** |  |  |  |  |  |  |
| Female (ref.) | 1.00 |  | 1.00 | — | 1.00 |  |
| Male | 1.03 (0.76–1.39) | 0.834 | 1.05 (0.75–1.45) | 0.772 | 0.77 (0.49 – 1.21) | 0.259 |
| **Age** |  |  |  |  |  |  |
| <60 years (ref.) | 1.00 |  | 1.00 | — | 1.00 |  |
| ≥60 years | **0.73 (0.55–0.98)** | **0.042** | 0.79 (0.57–1.08) | 0.139 | 0.88 (0.54 – 1.43) | 0.606 |
| **Educational level** |  |  |  |  |  |  |
| Primary or less (ref.) | — | — | 1.00 | — | 1.00 |  |
| Secondary or higher | — | — | 1.10 (0.81–1.48) | 0.540 | 1.03 (0.66 – 1.62) | 0.880 |
| **Duration of diabetes** |  |  |  |  |  |  |
| <10 years (ref.) | 1.00 |  | 1.00 | — | 1.00 |  |
| ≥10 years | **2.53 (1.80 – 3.56)** | **<0.001** | **2.28 (1.59–3.26)** | **<0.001** | 1.85 (1.09 – 3.14) | **0.021** |
| Hypertension |  |  |  |  |  |  |
| No (ref.) | 1.00 | — | 1.00 | — | 1.00 |  |
| Yes | 0.98 (0.71–1.435) | 0.920 | 0.99 (0.71 – 1.39) | 0.972 | 1.03 (0.63 – 1.67) | 0.908 |
| BMI |  |  |  |  |  |  |
| <30 kg/m^2^ (ref.) | — | — | 1.00 | — | 1.00 |  |
| ≥30 kg/m^2^ | — | — | 0.99 (0.73–1.37) | 0.983 | 1.16 (0.74 – 1.84) | 0.511 |
| **Diabetes treatment regimen** |  |  |  |  |  |  |
| None | 0.96 (0.64-1.46) | 0.874 | 1.03 (0.66 – 1.62) | 0.886 | 1.17 (0.61 – 1.45) | 0.656 |
| Only OADs (ref.) | 1.00 |  | 1.00 |  | 1.00 |  |
| Insulin only | **5.51 (3.36 – 9.702)** | **<0.001** | **5.35 (3.21 – 8.91)** | **<0.001** | **4.51 (2.21 – 9.27)** | **<0.001** |
| Insulin plus OADs | **4.91 (2.86 – 8.43)** | **<0.001** | **4.98 (2.86 – 8.69)** | **<0.001** | **4.62 (2.37 – 9.02)** | **<0.001** |
| Triglycerides |  |  |  |  |  |  |
| Normal (ref.) | — | — | — | — | 1.00 |  |
| Elevated | — | — | — | — | **1.88 (1.24 – 2.87)** | **0.003** |
| eGFR |  |  |  |  |  |  |
| ≥60 mL/min/1.73 m^2^ (ref.) | — | — | — | — | 1.00 |  |
| <60 mL/min/1.73 m^2^ | — | — | — | — | 0.50 (0.22 – 1.11) | 0.091 |

Results correspond to generalized estimating equation (GEE) models with a binomial distribution, logit link, exchangeable correlation structure, and robust standard errors. This analysis represents a sensitivity analysis in which no “missing data” category was created for variables with missing values; instead, a complete-case analysis was performed. Model 1 included only variables without missing data (n = 741); Model 2 additionally incorporated variables with <10% missing data (educational level and BMI; n = 665); and Model 3 further added variables with up to 35% missing data (triglycerides and estimated glomerular filtration rate; n = 312). Odds ratios (ORs) are presented with their 95% confidence intervals (95% CI) and p-values. An OR <1 indicates a lower probability of poor glycemic control, whereas an OR >1 indicates a higher probability.

BMI: body mass index; OAD: Oral antidiabetic drugs only; eGFR:Estimated Glomerular filtration rate
