# Supplementary material for: Longitudinal changes in glycemic control and associated factors in patients with type 2 diabetes mellitus in a public referral hospital in Peru
Source: PLoS One. 2026 Apr 6;21(4):e0346081. doi: 10.1371/journal.pone.0346081 (PMC13052837; doi:10.1371/journal.pone.0346081)
Supplement: S5 Table — (DOCX) [file pone.0346081.s009.docx]

**S5 Table. Median individual change in HbA1c from baseline to final evaluation and differences in medians across demographic, clinical, treatment, and laboratory subgroups**

|  | Individual HbA1c change  Median [p25–p75] | Difference in medians of individual HbA1c change  Median (95% CI) | p valueᶜ | Cohen’s d (95% CI) |
| --- | --- | --- | --- | --- |
| *Demographic variables* |  |  |  |  |
| Age |  |  |  |  |
| <60 years | 0.00 [-0.85 to 1.50] | 0 (-0.09 to 0.09) | 1.000 | -5.03 (-19.7 to 9.70) |
| ≥60 years | 0.00 [-0.60 to 1.20] |  |  |  |
| Sex |  |  |  |  |
| Female | 0.00 [-0.69 to 1.40] | 0 (-0.06 to 0.06) | 1.000 | 5.92 (-9.75 to 21.6) |
| Male | 0.00 [-0.59 to 1.20] |  |  |  |
| Educational level |  |  |  |  |
| Primary or less | 0.00 [-0.69 to 1.40] | 0 (-0.08 to 0.08) | 1.000 | 3.29 (-11.8 to 18.4) |
| Secondary or higher | 0.00 [-0.69 to 1.20] |  |  |  |
| Unknown | 0.00 [-0.59 to 1.30] |  |  |  |
| *Pathological history* |  |  |  |  |
| Hypertension |  |  |  |  |
| No | 0.00 [-0.69 to 1.40] | 0 (-0.14 to 0.14) | 1.000 | -12.6 (-29.4 to 4.1) |
| Yes | 0.00 [-0.69 to 1.20] |  |  |  |
| Tuberculosis |  |  |  |  |
| No | 0.00 [-0.60 to 1.40] | 0 (-0.64 to 0.64) | 1.000 | -34.3 (-74.3 to 5.6) |
| Yes | 0.00 [-1.70 to 0.59] |  |  |  |
| Duration of diabetes |  |  |  |  |
| <10 years | 0.00 [-0.60 to 1.40] | 0 (-0.08 to 0.08) | 1.000 | 5.66 (-10.2 to 21.6) |
| ≥10 years | 0.00 [-1.90 to 1.43] |  |  |  |
| *Clinical evaluation* |  |  |  |  |
| Abdominal obesity |  |  |  |  |
| No | 0.00 [-0.40 to 1.20] | 0 (-0.21 to 0.21) | 1.000 | -8.53 (-29.2 to 12.2) |
| Yes | 0.00 [-1.00 to 1.30] |  |  |  |
| No data | 0.00 [-0.60 to 1.40] |  |  |  |
| Obesity |  |  |  |  |
| BMI<30 kg/m^2^ | 0.00 [-0.60 to 1.40] | 0 (-0.08 to 0.08) | 1.000 | -14.6 (-30.1 to 1.00) |
| BMI ≥30 kg/m^2^ | 0.00 [-0.69 to 1.20] |  |  |  |
| Unknown | 0.65 [-0.40 to 3.75] |  |  |  |
| *Diabetes medication* |  |  |  |  |
| **Diabetes treatment regimen** |  |  |  |  |
| None | 0.05 [-0.19 to 1.60] | 0 (-0.03 to 0.03) | 1.000 |  |
| Onliy OADs (ref.) | 0.00 [-0.60 to 1.10] |  |  |  |
| Insulin only | 0.00 [-1.00 to 1.80] |  |  |  |
| Insulin plus oral antidiabetic drugs | 0.00 [-1.20 to 2.60] |  |  |  |
| *Laboratory variables* |  |  |  |  |
| Hypertriglyceridemia |  |  |  |  |
| No | 0.00 [-0.69 to 1.40] | 0 (-0.11 a 0.11) | 1.000 | 1.87 (-15.6 to 19.4) |
| Yes | 0.00 [-0.50 to 1.40] |  |  |  |
| No data | 0.00 [-0.90 to 1.33] |  |  |  |
| eGFR <60 mL/min/1.73 m^2^ |  |  |  |  |
| No | 0.00 [-0.69 to 1.43] | 0 (-0.28 to 0.28) | 1.000 | 7.28 (-35.6 to 21.0) |
| Yes | 0.00 [-1.30 to 1.80] |  |  |  |
| Missing data | 0.00 [-0.40 to 1.00] |  |  |  |
| Microalbuminuria |  |  |  |  |
| No | 0.00 [-0.59 to 1.40] | 0 (-0.23 to 0.23) | 1.000 | 7.97 (-23.6 to 39.5) |
| Yes | 0.00 [-0.50 to 1.40] |  |  |  |
| Missing data | 0.00 [-0.79 to 1.33] |  |  |  |
| Baseline HbA1c |  |  |  |  |
| < 7% | 0.00 [-0.90 to 0.19] | 0.19 (-0.44 to 0.04) | 0.106 | 67.2 (51.4 to 82.8) |
| ≥ 7% | 0.19 [-0.40 to 2.60] |  |  |  |

ᶜ Obtained from quantile regression with robust variance; H₀: difference in medians = 0.

Individual HbA1c changes are expressed as medians [p25–p75]. Differences in medians were evaluated using the Wilcoxon signed-rank test. Effect size was estimated using Cohen’s d with 95% CI. BMI: body mass index; OAD: Oral antidiabetic drugs only ; eGFR:Estimated Glomerular filtration rate
