# Supplementary material for: Longitudinal changes in glycemic control and associated factors in patients with type 2 diabetes mellitus in a public referral hospital in Peru
Source: PLoS One. 2026 Apr 6;21(4):e0346081. doi: 10.1371/journal.pone.0346081 (PMC13052837; doi:10.1371/journal.pone.0346081)
Supplement: S6 Table — (DOCX) [file pone.0346081.s010.docx]

**S6 Table. Individual change in HbA1c from baseline to final evaluation categorized in the overall population according to demographic, clinical, therapeutic, and laboratory factors**

|  | Δ A1c  < -0.5 | Δ A1c  -0.5 a +0.5 | Δ A1c  >0.5 | P value |
| --- | --- | --- | --- | --- |
| General | 210 (28.3) | 267 (36.0) | 230 (46.5) |  |
| *Demographic variables* |  |  |  |  |
| Age |  |  |  |  |
| <60 years | 87 (29.8) | 96 (32.9) | 109 (37.3) | 0.353 |
| ≥60 years | 123 (27.4) | 171 (38.1) | 155 (34.5) |  |
| Sex |  |  |  |  |
| Female | 150 (29.1) | 173 (33.5) | 193 (37.4) | 0.091 |
| Male | 60 (26.7) | 94 (41.8) | 71 (31.6) |  |
| Educational level (n=672) |  |  |  |  |
| Primary or less | 100 (28.6) | 121 (34.6) | 129 (36.8) | 0.654 |
| Secondary or higher | 92 (28.6) | 121 (37.6) | 109 (33.9) |  |
| *Pathological history* |  |  |  |  |
| Duration of diabetes |  |  |  |  |
| <10 years | 150 (28.4) | 188 (35.5) | 191 (36.1) | 0.885 |
| ≥10 years | 60 (28.3) | 79 (37.3) | 73 (34.4) |  |
| Hypertension |  |  |  |  |
| No | 153 (27.4) | 207 (37.0) | 199 (35.6) | 0.500 |
| Yes | 57 (31.3) | 60 (32.9) | 65 (35.7) |  |
| Tuberculosis |  |  |  |  |
| No | 199 (27.8) | 260 (36.3) | 257 (35.9) | 0.210 |
| Yes | 11 (44.0) | 7 (28.0) | 7 (28.0) |  |
| *Clinical evaluation* |  |  |  |  |
| Abdominal obesity (n=368) |  |  |  |  |
| No | 37 (24.0) | 67 (43.5) | 50 (32.5) | **0.028** |
| Yes | 70 (32.7) | 65 (30.4) | 79 (36.9) |  |
| Obesity (n=733) |  |  |  |  |
| BMI <30 kg/m^2^ | 136 (27.3) | 187 (37.5) | 176 (35.3) | 0.480 |
| BMI ≥30 kg/m^2^ | 72 (30.8) | 78 (33.3) | 84 (35.9) |  |
| *Diabetes medication* |  |  |  |  |
| **Diabetes treatment regimen** |  |  |  |  |
| None | 17 (20.5) | 31 (37.4) | 35 (42.2) | 0.161 |
| OADs (ref.) | 131 (29.1) | 173 (38.4) | 146 (32.4) |  |
| Insulin only | 37 (29.1) | 42 (33.1) | 48 (37.8) |  |
| Insulin plus OADs | 25 (30.9) | 21 (25.9) | 35 (43.2) |  |
| *Laboratory* |  |  |  |  |
| Hypertriglyceridemia (n=500) |  |  |  |  |
| No | 74 (30.1) | 89 (36.2) | 83 (33.7) | 0.443 |
| Yes | 64 (25.2) | 102 (40.2) | 88 (34.7) |  |
| eGFR <60 mL/min/1.73 m^2^ (n=482) |  |  |  |  |
| No | 125 (29.2) | 136 (31.8) | 167 (39.0) | 0.273 |
| Yes | 21 (38.9) | 17 (31.5) | 16 (29.6) |  |
| Microalbuminuria (n=208) |  |  |  |  |
| No | 45 (28.7) | 59 (37.6) | 53 (33.8) | 0.874 |
| Yes | 13(25.5) | 21 (41.2) | 17 (33.3) |  |
| Baseline HbA1c |  |  |  |  |
| < 7% | 87 (35.2) | 126 (51.0) | 34 (13.8) | **<0.001** |
| ≥ 7% | 123 (24.9) | 141 (28.5) | 230 (46.6) |  |

BMI: body mass index; OAD: Oral antidiabetic drugs only ; eGFR:Estimated Glomerular filtration rate
