# Supplementary material for: Longitudinal changes in glycemic control and associated factors in patients with type 2 diabetes mellitus in a public referral hospital in Peru
Source: PLoS One. 2026 Apr 6;21(4):e0346081. doi: 10.1371/journal.pone.0346081 (PMC13052837; doi:10.1371/journal.pone.0346081)
Supplement: S7 Table — (DOCX) [file pone.0346081.s011.docx]

**S7 Table. Proportion of patients with a decrease or increase ≥0.5% in HbA1c according to demographic, clinical, and treatment characteristics, stratified by baseline HbA1c (<7% and ≥7%)**

|  | Baseline HbA1c <7%% | | | Baseline HbA1c ≥7% | | |
| --- | --- | --- | --- | --- | --- | --- |
|  | No disminuye  0.5% | Disminuye 0.5% | Valor p | No aumenta  0.5% | Aumenta 0.5% | Valor p |
| *Demographics* |  |  |  |  |  |  |
| Age |  |  |  |  |  |  |
| <60 years | 53 (57.6) | 39 (42.4) | 0.069 | 76 (82.6) | 16 (17.4) | 0.203 |
| ≥60 years | 107 (69.0) | 48 (31.0) |  | 137 (88.4) | 18 (11.6) |  |
| Sex |  |  |  |  |  |  |
| Female | 117 (65.7) | 61 (34.3) | 0.615 | 151 (84.8) | 27 (15.2) | 0.304 |
| Male | 43 (62.3) | 26 (37.7) |  | 62 (89.9) | 7 (10.1) |  |
| Educational level |  |  |  |  |  |  |
| Primary or less | 77 (63.6) | 44 (36.3) | 0.819 | 106 (87.6) | 15 (12.4) | 0.555 |
| Secondary or higher | 69 (65.1) | 37 (34.9) |  | 90 (84.9) | 16 (15.1) |  |
| *Pathological history* |  |  |  |  |  |  |
| Duration of diabetes |  |  |  |  |  |  |
| <10 years | 135 (65.5) | 71 (34.5) | 0.577 | 174 (84.5) | 32 (15.5) | 0.083 |
| ≥10 years | 25 (61.0) | 16 (39.0) |  | 39 (95.1) | 2 (4.9) |  |
| Hypertension |  |  |  |  |  |  |
| No | 118 (66.3) | 60 (33.7) | 0.423 | 153 (85.9) | 25 (14.0) | 0.999 |
| Yes | 42 (60.9) | 27 (39.1) |  | 60 (87.0) | 9 (13.0) |  |
| Tuberculosis |  |  |  |  |  |  |
| No | 157 (65.4) | 83 (34.6) | 0.246 | 208 (86.7) | 32 (13.3) | 0.248 |
| Yes | 3 (42.9) | 4 (57.1) |  | 5 (71.4) | 2 (28.6) |  |
| *Clinical evaluation* |  |  |  |  |  |  |
| Abdominal obesity |  |  |  |  |  |  |
| No | 35 (70.0) | 15 (30.0) | 0.218 | 43 (86.0) | 7 (14.0) | 0.999 |
| Yes | 45 (59.2) | 31 (40.8) |  | 64 (84.2) | 12 (15.8) |  |
| Obesity |  |  |  |  |  |  |
| BMI <30 kg/m^2^ | 102 (67.6) | 49 (32.4) | 0.319 | 129 (85.4) | 22 (14.6) | 0.715 |
| BMI ≥30 kg/m^2^ | 57 (61.3) | 36 (38.7) |  | 81 (87.1) | 12 (12.9) |  |
| *Diabetes medication* |  |  |  |  |  |  |
| **Diabetes treatment regimen** |  |  |  |  |  |  |
| None | 24 (70.6) | 10 (29.4) | 0.362 | 28 (82.4) | 6 (17.7) | 0.756 |
| Oral antidiabetic drugs only (ref.) | 125 (65.1) | 67 (34.9) |  | 165 (85.9) | 27 (14.1) |  |
| Insulin only | 8 (61.5) | 4 (38.5) |  | 12 (92.3) | 1 (7.7) |  |
| Insulin plus oral antidiabetic drugs | 3 (37.5) | 5 (62.5) |  | 8 (100.0) | 0 (0.0) |  |
| *Laboratory* |  |  |  |  |  |  |
| Hypertriglyceridemia |  |  |  |  |  |  |
| No | 65 (65.7) | 34 (34.3) | 0.759 | 87 (87.9) | 12 (12.1) | 0.707 |
| Yes | 45 (63.4) | 26 (36.6) |  | 61 (85.9) | 10 (14.1) |  |
| eGFR <60 mL/min/1.73 m^2^ |  |  |  |  |  |  |
| No | 84 (60.4) | 55 (39.6) | 0.455 | 114 (82.0) | 25 (18.0) | 0.372 |
| Yes | 12 (52.2) | 11 (47.8) |  | 21 (91.3) | 2 (8.7) |  |
| Microalbuminuria |  |  |  |  |  |  |
| No | 28 (60.9) | 18 (39.1) | 0.778 | 41 (89.1) | 5 (10.9) | 0.676 |
| Yes | 12 (66.7) | 6 (33.3) |  | 15 (83.3) | 3 (16.7) |  |

A change of ≥0.5% in HbA1c was considered clinically relevant. P-values were obtained using the chi-square test or Fisher’s exact test, as appropriate, within each category of the independent variable.
