# Supplementary material for: Longitudinal changes in glycemic control and associated factors in patients with type 2 diabetes mellitus in a public referral hospital in Peru
Source: PLoS One. 2026 Apr 6;21(4):e0346081. doi: 10.1371/journal.pone.0346081 (PMC13052837; doi:10.1371/journal.pone.0346081)
Supplement: Attachment — Submitted filename: Response to reviewers.docx [file pone.0346081.s013.docx]

**Response to Journal Requirements**

We thank the editorial office for the additional guidance regarding the journal requirements. We have carefully reviewed these points and revised the submission accordingly to ensure full compliance with the PLOS ONE formatting, data sharing, ethics, and figure preparation guidelines. Detailed responses to each item are provided below.

**1. Please ensure that your manuscript meets PLOS ONE's style requirements, including those for file naming. The PLOS ONE style templates can be found at https://journals.plos.org/plosone/s/file?id=wjVg/PLOSOne_formatting_sample_main_body.pdf and https://journals.plos.org/plosone/s/file?id=ba62/PLOSOne_formatting_sample_title_authors_affiliations.pdf**

We reviewed the manuscript and ensured that it complies with the PLOS ONE formatting and style requirements, including file naming and manuscript structure according to the journal’s guidelines.

**2. The ethical approval number(s) listed in the manuscript and/or submission metadata does not match the approval number on the ethical approval document you provided. Please ensure that all approval numbers are correct.**

Thank you for pointing this out. The discrepancy was due to a typographical error in the manuscript, where ethical approval number was mistakenly written as 500-CIEI-CIENTÍFICA-2024 instead of 526-CIEI-CIENTÍFICA-2024. The manuscript has now been corrected to match the ethics approval document. The project (PRE-15-2024-00188) was approved by the Institutional Research Ethics Committee of Universidad Científica del Sur under Certificate No. 526-CIEI-CIENTÍFICA-2024, and also by the Institutional Ethics Committee of Hospital María Auxiliadora under Unique Registration Code HMA/CIEI/047/2024.

**3. We note that the grant information you provided in the ‘Funding Information’ and ‘Financial Disclosure’ sections do not match. When you resubmit, please ensure that you provide the correct grant numbers for the awards you received for your study in the ‘Funding Information’ section.**

Thank you for pointing this out. The information entered in the **Funding Information section of the submission system is correct**. The discrepancy occurred in the **Financial Disclosure field of the system**, where the internal project code was mistakenly entered instead of the official funding resolution. At the current stage of the revision process, the Financial Disclosure field in the system is **not editable by the authors**. Therefore, we provide below the **correct wording that should appear in the Financial Disclosure statement**, consistent with the Funding Information:

Financial support for this study was partially provided by Universidad Científica del Sur (UCSUR) through the Beca Cabieses – Proyectos de Tesis de Pregrado program (grant number **RD No. 004-DGIDI-CIENTIFICA-2024**), awarded to the authors RBCM and JMQE. The official website of the funder is <https://www.cientifica.edu.pe>. The funder had no role in the study design, data collection and analysis, interpretation of results, decision to publish, or preparation of the manuscript. If the manuscript is accepted for publication, the university may provide support to cover the article processing charge (APC); this potential support will not influence any aspect of the editorial process.

We kindly ask the editorial office to update the Financial Disclosure field in the system if necessary so that it matches the Funding Information.

**4. Please upload a new copy of Figure 2 and Supporting Information Figure 1 and 2 as the detail is not clear. Please follow the link for more information: https://journals.plos.org/plosone/s/figures**

The requested figures (Figure 2 and Supporting Figures S1–S2) have been updated and re-uploaded with improved resolution and clarity in accordance with the PLOS ONE figure guidelines.

**5. We note that there is identifying data in the Supporting Information file < S1 File.xlsx>. Due to the inclusion of these potentially identifying data, we have removed this file from your file inventory. Prior to sharing human research participant data, authors should consult with an ethics committee to ensure data are shared in accordance with participant consent and all applicable local laws. Data sharing should never compromise participant privacy. It is therefore not appropriate to publicly share personally identifiable data on human research participants. The following are examples of data that should not be shared: -Name, initials, physical address -Ages more specific than whole numbers -Internet protocol (IP) address -Specific dates (birth dates, death dates, examination dates, etc.) -Contact information such as phone number or email address -Location data -ID numbers that seem specific (long numbers, include initials, titled “Hospital ID”) rather than random (small numbers in numerical order) Data that are not directly identifying may also be inappropriate to share, as in combination they can become identifying. For example, data collected from a small group of participants, vulnerable populations, or private groups should not be shared if they involve indirect identifiers (such as sex, ethnicity, location, etc.) that may risk the identification of study participants. Additional guidance on preparing raw data for publication can be found in our Data Policy (https://journals.plos.org/plosone/s/data-availability#loc-human-research-participant-data-and-other-sensitive-data) and in the following article: http://www.bmj.com/content/340/bmj.c181.long. Please remove or anonymize all personal information (<specific identifying information in file to be removed>), ensure that the data shared are in accordance with participant consent, and re-upload a fully anonymized data set. Please note that spreadsheet columns with personal information must be removed and not hidden as all hidden columns will appear in the published file.**

Thank you for this observation. We carefully reviewed the dataset to ensure compliance with PLOS data sharing policies and participant confidentiality requirements. All potential identifiers were removed from the dataset prior to resubmission. In particular, the internal record identifier column was deleted, and we verified that the dataset does not contain personal identifiers such as names, medical record numbers, contact information, dates, or location data. The fully anonymized dataset has now been re-uploaded as **S1 Dataset.xlsx**.

**6. If the reviewer comments include a recommendation to cite specific previously published works, please review and evaluate these publications to determine whether they are relevant and should be cited. There is no requirement to cite these works unless the editor has indicated otherwise.**

We reviewed the reviewer comments and confirmed that no specific recommendations to cite additional publications were made. No changes to the reference list were required.

**Response to Reviewers**

We thank the reviewers for their constructive comments and suggestions. We carefully considered all the points raised and revised the manuscript accordingly. Our responses to each comment are provided below.

**Reviewer #1**

**1. Abstract: Suggestions: Emphasize the clinical and public health relevance of the findings in the conclusion (e.g., implications for diabetes care in Peru)**

We appreciate this suggestion. The conclusion of the abstract has been revised to highlight the clinical and public health implications of our findings for diabetes management within the Peruvian public health system.

**2. Clarify that the reduction was statistically significant but clinically modest, to balance interpretation.**

We agree with the reviewer and have modified the conclusion of the abstract to explicitly state that although the reduction in poor glycemic control was statistically significant, the magnitude of the improvement was clinically modest.

**3. Consider shortening sentences for readability, especially in the Methods section**

The Methods section of the abstract has been revised to improve readability by splitting long sentences into shorter statements while preserving the methodological details.

**Reviewer #2**

**1. Abstract: Kindly clarify at the methods section, that patients with T2DM**

We thank the reviewer for this suggestion. The Methods section of the abstract has been revised to explicitly state that the study population consisted of adults diagnosed with type 2 diabetes mellitus (T2DM).

**2. Were changes in treatment assessed in this retrospective study? The authors make mention of not assessing adherence to treatment earlier however assessing changes in treatment could also help evaluate the factors associated with good or poor glycaemic control**

Changes in pharmacological treatment during follow-up were not systematically captured in the database used for this retrospective study. We have clarified this point in the limitations section, noting that treatment intensification or medication adjustments could influence glycemic control and should be considered in future studies.

**3. Kindly check referencing for Line 383-384**

Thank you for noting this. The citation was incorrectly placed on the following line. It has now been moved to appear before the final period of the sentence
